# Supplementary material for: Longitudinal change in executive function is associated with impaired top-down frontolimbic regulation during reappraisal in older adults
Source: Neuroimage. 2021 Jan 15;225:117488. doi: 10.1016/j.neuroimage.2020.117488 (PMC7779563; doi:10.1016/j.neuroimage.2020.117488)
Supplement: Supplementary file 1 [file mmc1.docx]

**Supplementary Material**

*Rate of change of longitudinal executive function (rLEF)*

rLEF was calculated by combining scores from verbal fluency category and trail-making tasks, collected longitudinally over a varying number of years. See figure A1 for a graphical representation of change in raw scores for each individual/task. The average time span between first and last data points was 6.9 years (std dev. = 3.1 years). The average number of time points was 2.97 (std dev. = 0.77) with a minimum of 2 and a maximum of 4. The average time between most recent test and imaging was 13 months, including four outliers (4.4, 4.7, 4.9 and 5.1 years) where the most recent test data was not usable. The mean slope of verbal fluency was 0.16 std dev. = 1.01) and trail making was -0.03 (std dev. = 0.144). These scores were normalised and combined in order to give a relative composite measure of changes in cognitive performance within the group.


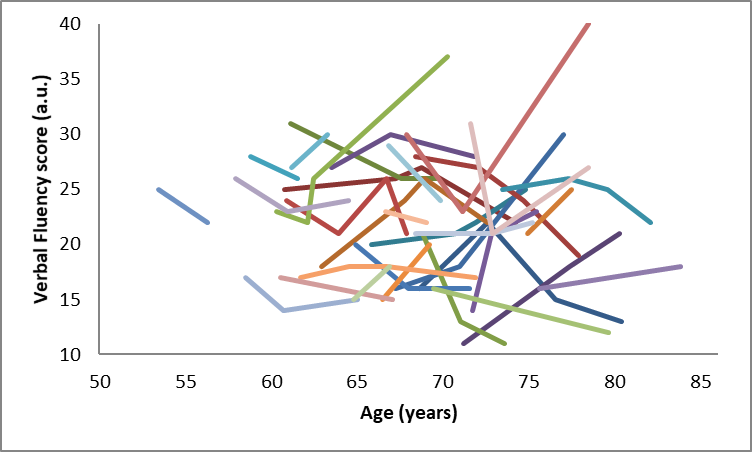

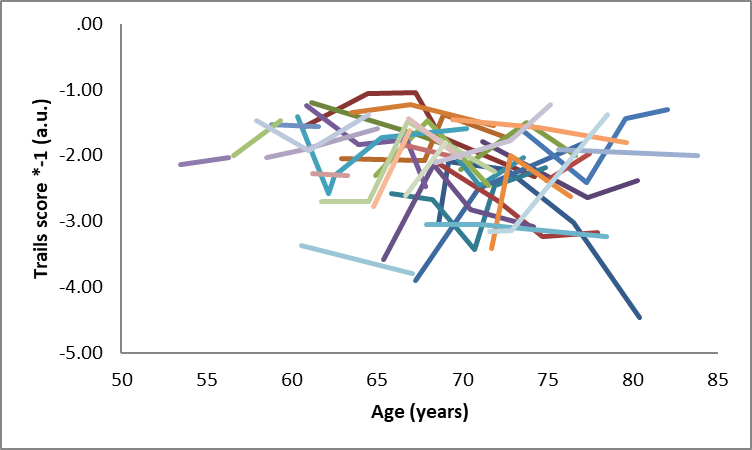


**A**

**B**

Figure S1: Graphs demonstrating the change in scores for each participant in the verbal fluency category (A) and trails tasks (B).

| **Table S1** |  |  |  |  |  |  |
| --- | --- | --- | --- | --- | --- | --- |
| A-priori PFC and amygdala regional activation patterns in response to stimuli presented in the emotion regulation task. **Participants with** **MMSE < 26 excluded** | |  |  |  |  |  |
|  |  |  |  |  |  |  |
| Contrast | Brain region | Voxels | Max Z | Location of max Z | | |
|  |  |  |  | x | y | z |
| Suppress > Passive | L VLPFC | 237 | 4.68 | -52 | 24 | 10 |
| Suppress > Passive | L VLPFC | 30 | 4.83 | -44 | 14 | 24 |
| Suppress > Passive | R VLPFC | 12 | 4.25 | 50 | 28 | -2 |
| Suppress > Passive | R VLPFC | 1 | 4.26 | 56 | 32 | -2 |
|  |  |  |  |  |  |  |
| Suppress > Passive x rLEF | R Amygdala | 7 | 4.25 | 24 | -2 | -18 |
|  |  |  |  |  |  |  |
| Enhance > Passive | L VLPFC | 1667 | 5.33 | -48 | 20 | 26 |
| Enhance > Passive | R VLPFC | 506 | 5.11 | 50 | 26 | -2 |
| Enhance > Passive | R VLPFC | 72 | 4.07 | 26 | 28 | -12 |
| Enhance > Passive | R VLPFC | 22 | 4.14 | 54 | 32 | 14 |
| Enhance > Passive | L VLPFC | 3 | 3.17 | -40 | 28 | 18 |
| Enhance > Passive | L VLPFC | 1 | 2.93 | -24 | 30 | -16 |
| Enhance > Passive | VMPFC | 130 | 4.40 | 2 | 40 | 4 |
| Enhance > Passive | VMPFC | 10 | 3.95 | 8 | 48 | -4 |
|  |  |  |  |  |  |  |
| Note: Corrected cluster for multiple comparisons at *p* < 0.05. Location of cluster's maximum Z are in MNI space. R = right; L = left. rLEF = the rate of change of longitudinal executive function. | | | | | | |
|  |  |  |  |  |  |  |
